# Morbidity burden of respiratory diseases attributable to ambient temperature: a case study in a subtropical city in China

**DOI:** 10.1186/s12940-019-0529-8

**Published:** 2019-10-24

**Authors:** Yiju Zhao, Zhao Huang, Shengyong Wang, Jianxiong Hu, Jianpeng Xiao, Xing Li, Tao Liu, Weilin Zeng, Lingchuan Guo, Qingfeng Du, Wenjun Ma

**Affiliations:** 1 Department of Respirator Medicine, The Fifth People’s Hospital of Dongguan, Dongguan, China; 2 The First Affiliated Hospital of Guangzhou University of Traditional Chinese Medicine, Guangzhou, China; 3 Medical College, Jinan University, Guangzhou, China; 4 School of Public Health, Guangdong Pharmaceutical University, Guangzhou, China; 5 Guangdong Provincial Institute of Public Health, Guangdong Provincial Center for Disease Control and Prevention, Guangzhou, 511430 China; 6 Nanhai Hospital of Southern Medical University, Foshan, China

**Keywords:** Respiratory disease, Moderate heat, Temperature, Disease burden

## Abstract

**Background:**

There are significant associations between ambient temperature and respiratory disease mortality. However, few studies have assessed the morbidity burdens of various respiratory diseases that are attributable to different temperature ranges in subtropical regions.

**Methods:**

Daily outpatient visits, weather variables, and air pollution data were collected from January 2013 to August 2017 in a hospital in Dongguan city. A standard time series quasi-Poisson regression with a distributed lag non-linear model (DLNM) was applied to estimate the associations between daily mean temperature and morbidity for total respiratory diseases, bronchiectasis, chronic obstructive pulmonary disease (COPD), and asthma. Attributable fractions were then calculated to quantify disease burden relative to different temperature components. Finally, we conducted stratified analysis by age group.

**Results:**

Both low and high temperatures were associated with an increased risk of morbidity secondary to respiratory diseases. Compared with the optimum temperature, the accumulated relative risk (RR) during the seven lag days was 1.13 with a 95% confidence interval (CI) of 1.01–1.26 for extreme heat and 1.02 (95% CI: 0.99–1.05) for extreme cold. Heat-related respiratory morbidity risk was higher than cold-related risk for the total population, but an opposite result was observed for the elderly. About 8.4% (95% CI: 2.8–13.3%) of respiratory morbidity was attributable to non-optimal temperatures, and moderate heat was responsible for most of the excess respiratory morbidity (7.5, 95% CI: 2.4–12.2%).

**Conclusions:**

We found that exposure to non-optimal temperatures increased the risk of respiratory morbidity in subtropical region, and moderate heat contributed to most of the temperature-related respiratory morbidities. This indicates a need for further examination of moderate, rather than extreme, heat in subtropical region.

## Background

Respiratory diseases, including chronic obstructive pulmonary disease (COPD), asthma, bronchiectasis, pneumonia, etc., have been implicated in more than 354 million deaths worldwide [1]. In recent years, respiratory diseases were responsible for 11% of all deaths in China [2]. Therefore, respiratory diseases are a significant public health problem, both around the world and in China.

The associations between ambient temperatures and human health have been widely studied, and growing evidence has revealed that exposure to ambient temperatures may increase the risks of a range of respiratory diseases, cardiovascular diseases, and other diseases [3–7]. Although most previous studies found significant relationships between ambient temperature and morbidity or mortality [8, 9], few have assessed the disease burden attributable to ambient temperatures [10–14]. Compared with relative risk (RR), disease burden may provide extra public health information by calculating attributable fraction (AF) and absolute excess numbers such as the attributable number (AN). AF and AN can quantify health burden due to specific temperature range exposure for specific diseases [15]. This is critical for planning and evaluating public health interventions and controls.

Also, most previous studies explored the associations between temperature and health outcomes, with a focus on mortality rather than morbidity. There are some limitations in the analysis of associations between climate change or variations and mortality: the actual death time and the reported time may be inconsistent. Moreover, mortality only reflects the most serious health outcomes of ambient temperature exposure. Compared with mortality, morbidity includes a broader range of health events such as outpatient visits, emergency room visits, and hospitalizations, which was more suitable to examine the acute effect of temperature exposure on health. A few studies have evaluated the effect of extreme temperature on morbidity [16–19]. However, less attention has been paid to the burden of respiratory disease in relationship to different temperature ranges, such as extreme heat, moderate heat, extreme cold and moderate cold.

In the present study, we aimed to examine the associations between ambient temperature and hospital outpatient visits for total respiratory diseases, bronchiectasis, COPD, and asthma in Dongguan City, China. We further quantified the morbidity burdens attributable to different temperature ranges by computing AF and AN. The findings from our study may help to provide a better understanding of ambient temperature exposure on respiratory morbidity burden.

## Methods

### Data

#### Hospital outpatient visits data

Dongguan, located in south China, is a subtropical city. Daily data on hospital outpatient visits for respiratory illness between January 2013 and August 2017 were collected from the computerized database of the Dongguan Fifth People’s Hospital, located in southwest Dongguan City. The hospital is one of the premier hospitals in Dongguan. Most patients in this study were local residents because of medical insurance policy restrictions and convenience. The specific causes of hospital outpatient visits were coded according to the tenth revision of the International Classification of Diseases (ICD-10) and included total respiratory diseases (J00–J99), bronchiectasis (J47), COPD (J40–J44), and asthma (J45–J46).

#### Meteorological and air pollution data

Daily meteorological data were collected from 2013 to 2017 from the China National Weather Data Sharing System (http://cdc.cma.gov.cn/home.do), including daily mean temperature and relative humidity. Weather data were gathered by a fixed-site station located at the center of Dongguan City.

To adjust for the effect of potential confounding variables, we obtained daily concentrations of air pollutants including Particulate matter with an aerodynamic diameter ≤ 10 μm (PM_10_), sulfur dioxide (SO_2_), O_3_, and nitrogen dioxide (NO_2_) from the Dongguan Environmental Monitoring Center. These levels were measured by averaging values obtained from five fixed air monitoring stations (Additional file 1: Figure S1).

#### Statistical model

We used a two-stage analysis approach. During the first stage, we applied a standard time series quasi-Poisson regression with a distributed lag non-linear model (DLNM) to estimate the associations between daily mean temperature and morbidity of total and specific respiratory-related outpatient visits. Meanwhile, we conducted stratified analyses by age group to observe the potential effects of age on temperature-respiratory morbidity relationships.

The exposure-response relationship was modeled using a quadratic B-spline for temperature with three internal knots placed at equal temperature interval distributions, and the lag-response curve used a natural cubic spline with an intercept and three internal knots placed at equally spaced values along the log scale [14]. We used this stratified strategy to control for seasonality and long-term trends by the natural cubic spline of the year with 4 degrees of freedom and month with 3 degrees of freedom [20]. To control for time-varying confounders, we included relative humidity, air pollutants, dummy variables for day of the week, and public holidays in the model. We also defined three degrees of freedom for relative humidity, PM_10_, SO_2,_ NO_2,_ and O_3,_ consistent with several previous studies. Akaike’s Information Criterion for quasi-Poisson (Q-AIC) was used to evaluate the model fits, which was subsequently applied to other studies [21–23]. In all the models, we used seven days as the maximum lag to adequately assess cold- and heat-related effects on hospital outpatient visits. The model was:

$$ \log \left[E\left({Y}_t\right)\right]=\alpha +\beta {\mathrm{T} emp}_{t,l}+\gamma Strata+ ns\left({RH}_t,3\right)+ ns\left({PM}_{10t},3\right)+ ns\left({SO}_{2t},3\right)+ ns\left({NO}_{2t},3\right)+ ns\left({O}_{3t},3\right)+\delta {DOW}_t+\varepsilon {Holiday}_t $$

where *t* is the day of the study period; E(*Y*_*t*_) presents the expected daily counts for hospital outpatient visits for respiratory diseases on day *t*; α is the intercept; *Temp*_*t*, *l*_ is the cross-basis matrix obtained by modeling the DLNM to temperature; *l* is the lag days. Strata refers two categorical variables of year and calendar month that were used to control for long-term trends and seasonality; ns(.) means the natural cubic spline function for nonlinear variables; *df* is the degrees of freedom; RH is the relative humidity; DOW is a categorical variable for controlling for the day of the week. Holiday is a binary variable. If the day *t* is a public holiday the value of 1 otherwise 0; *β*, γ, δ, ε are vectors of coefficients for corresponding terms.

The temperature of the minimum morbidity (MMT), characterized by the lowest risk of respiratory hospital outpatient visits, was derived from the lowest point of overall cumulative exposure-response curve. We referred to the value as the optimum temperature (centering value) for fitting the exposure–lag–response relationship in each model. Extreme cold and heat were defined temperatures at the 5th percentile or less (extreme cold), and at the 95th percentile or above (extreme heat). We calculated the RRs and 95% CIs of respiratory morbidity at the 5th and 95th percentile of temperature distribution, referenced to the optimum temperature for representing the effects of extreme cold and heat.

#### Estimation of AR

During the second stage, we used a backward perspective strategy to quantify the burden of respiratory outpatient visits due to temperature exposures. This approach examined from current risk to past exposures, and has been described in detail elsewhere [14]. Backward standpoint assumes that the risk at time *t* can be attributed to a series of past exposure events. The backward attributable fraction AF_*x,t,*_ and number AN_*x,t*_ at time *t* can be expressed as:

$$ {AF}_{x,t}=1-\mathit{\exp}\left(-\sum \limits_{l={l}_0}^L{\beta}_{x_{t-l}},l\right) $$

$$ {AN}_{x,t}={AF}_{\mathrm{x},t}\bullet {n}_t $$

Here, *n*_*t*_ is the number of cases at time *t*. The risk at time *t* is associated with lagged exposures at times *t − l.* AN_*x,t*_ and AF_*x,t*_ are the number of cases and the related fraction at time *t* attributable to past exposures to *x* in the period *t − l*_*0*_,. .., *t − L*.

The optimum temperature was also regarded as the reference for computing the fractions and the numbers of excess outpatient visits attributable to the temperatures. We further divided the temperature distribution into the extreme cold and heat, and the moderate cold and heat. We defined “moderate temperatures” as the ranges between the optimum temperature and the 5th and 95th percentiles (Additional file 2: Figure S2). We then calculated the health burden attributable to extreme and moderate temperatures with reference to the optimum temperature. The estimation of empirical CIs for attributable fractions and numbers were obtained through Monte Carlo simulations [24, 25], by simulating 5000 random samples. All the statistical analyses were performed using the R software (version 3.4.4) with the “*dlnm*” package.

## Results

### The characteristics of the sample

**Fig. 1:**
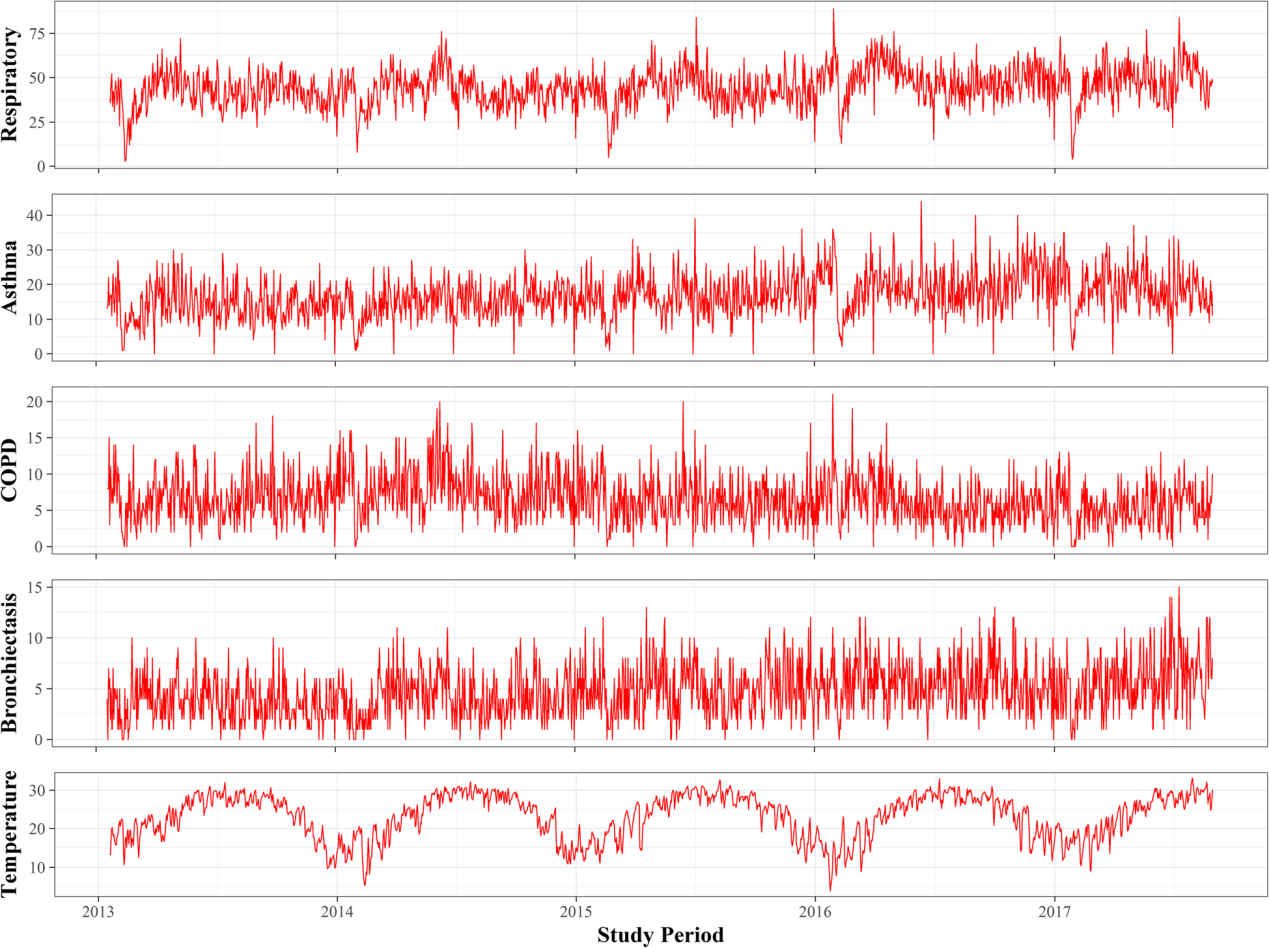
Time-series plots for daily hospital outpatient visits (counts/day) for total respiratory diseases, asthma, chronic obstructive pulmonary disease (COPD), bronchiectasis, and daily mean temperature (°C)

Table 1 summarizes morbidity, weather, and air pollution data in Dongguan City. During the study period, there were 75,015 hospital outpatient visits related to respiratory diseases. Of these, there were 11,068 cases of COPD, 28,059 cases of asthma, and 7991 cases of bronchiectasis. On average, there were 44 respiratory-related hospital outpatient visits. Of these, 17 were related to asthma, 7 were related to COPD, and 5 were related to bronchiectasis. The daily mean temperature was 23.2 °C (3.8–33.2 °C), and the relative humidity was 77.5% with a range of 22.0–100.0%. The daily mean concentration was 56.6 μg/m^3^ for PM_10_, 15.9 μg/m^3^ for SO_2_, 38.8 μg/m^3^ for NO_2_, and 62.0 μg/m^3^ for O_3_. Figure 1 depicts a time series plot of daily mean temperatures and hospital outpatient visits for total and specific-respiratory diseases.

**Table 1:** Summary statistics of daily hospital outpatient visits, weather conditions, and air pollutants in Dongguan (2013–2017)

Variable	Mean ± SD	Percentile				
		Min	P25	P50	P75	Max
Daily outpatients						
Respiratory	44 ± 11	3	38	44	52	89
asthma	17 ± 6	0	12	16	21	44
COPD	7 ± 3	0	4	6	8	21
Bronchiectasis	5 ± 3	0	3	5	6	15
Meteorological factors						
Temperature (°C)	23.2 ± 5.8	3.8	19.0	24.8	28.0	33.2
Relative humidity (%)	77.5 ± 13.4	22.0	70.0	79.0	87.0	100.0
Air pollution (μg/m^3^)						
PM_10_	56.6 ± 28.3	7.7	35.5	49.4	71.0	217.9
SO_2_	15.9 ± 8.4	3.1	10.0	13.8	19.3	60.3
NO_2_	38.8 ± 16.3	9.0	27.4	36.0	47.0	137.3
O_3_	62.0 ± 34.7	5.2	37.9	55.5	79.8	304.9

### Morbidity risk of temperature on respiratory diseases

**Fig. 2:**
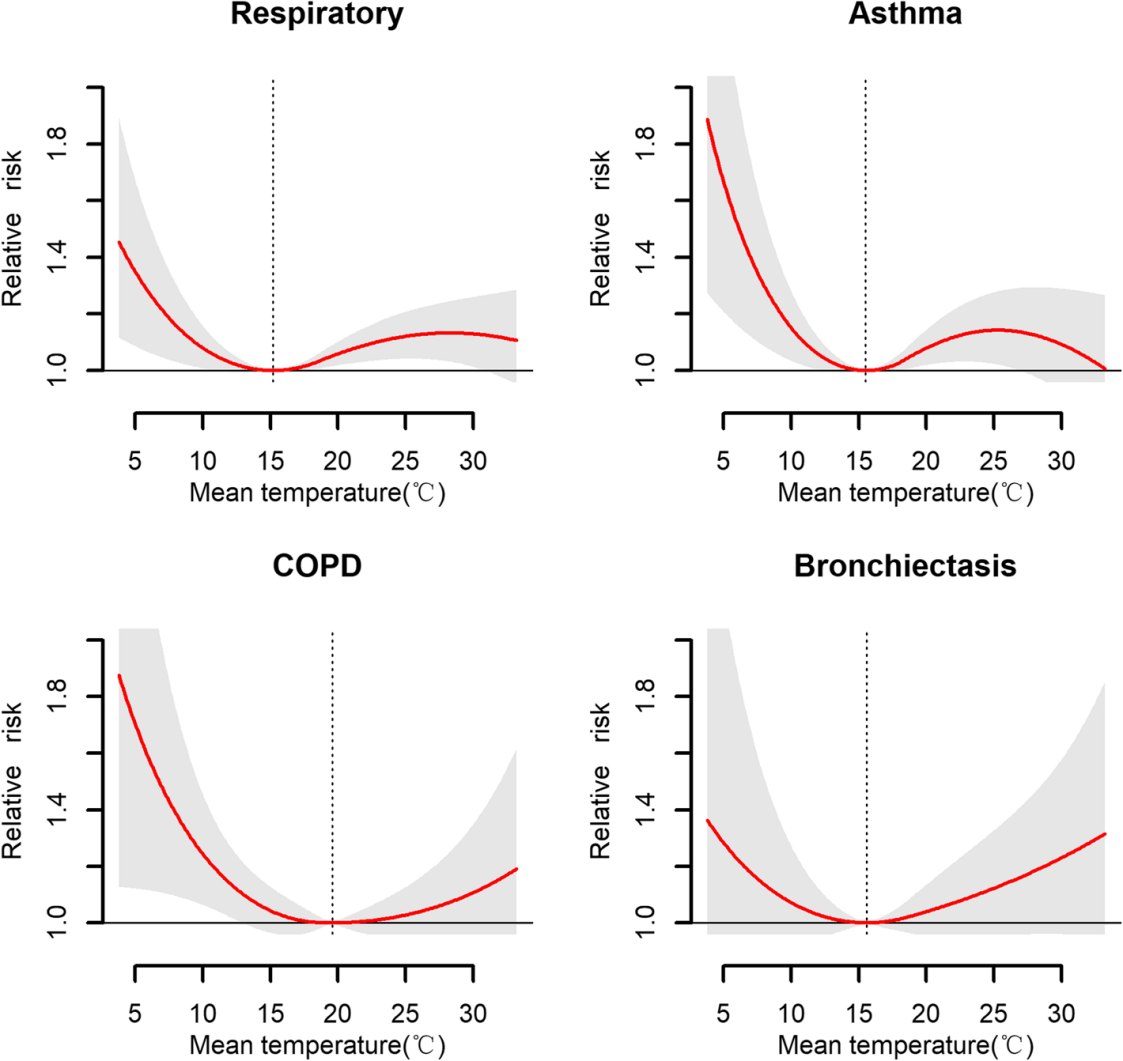
The cumulative associations between mean temperature and daily hospital outpatient visits for total respiratory diseases, asthma, COPD, and bronchiectasis over a lag of 0–7 days. The shaded grey is the 95% CI, and the dashed lines show the optimal temperatures of 15.20 °C, 15.50 °C, 19.60 °C, and 15.60 °C. Respiratory indicates respiratory disease; COPD means chronic obstructive pulmonary disease

Figure 2 shows the temperature–morbidity curves for total and specific respiratory diseases. The associations between temperature and morbidity of respiratory diseases are U or inverse J-shaped. The risks of temperature on total respiratory diseases, asthma, and COPD were significantly higher at low temperatures than at high temperatures.

**Fig. 3:**
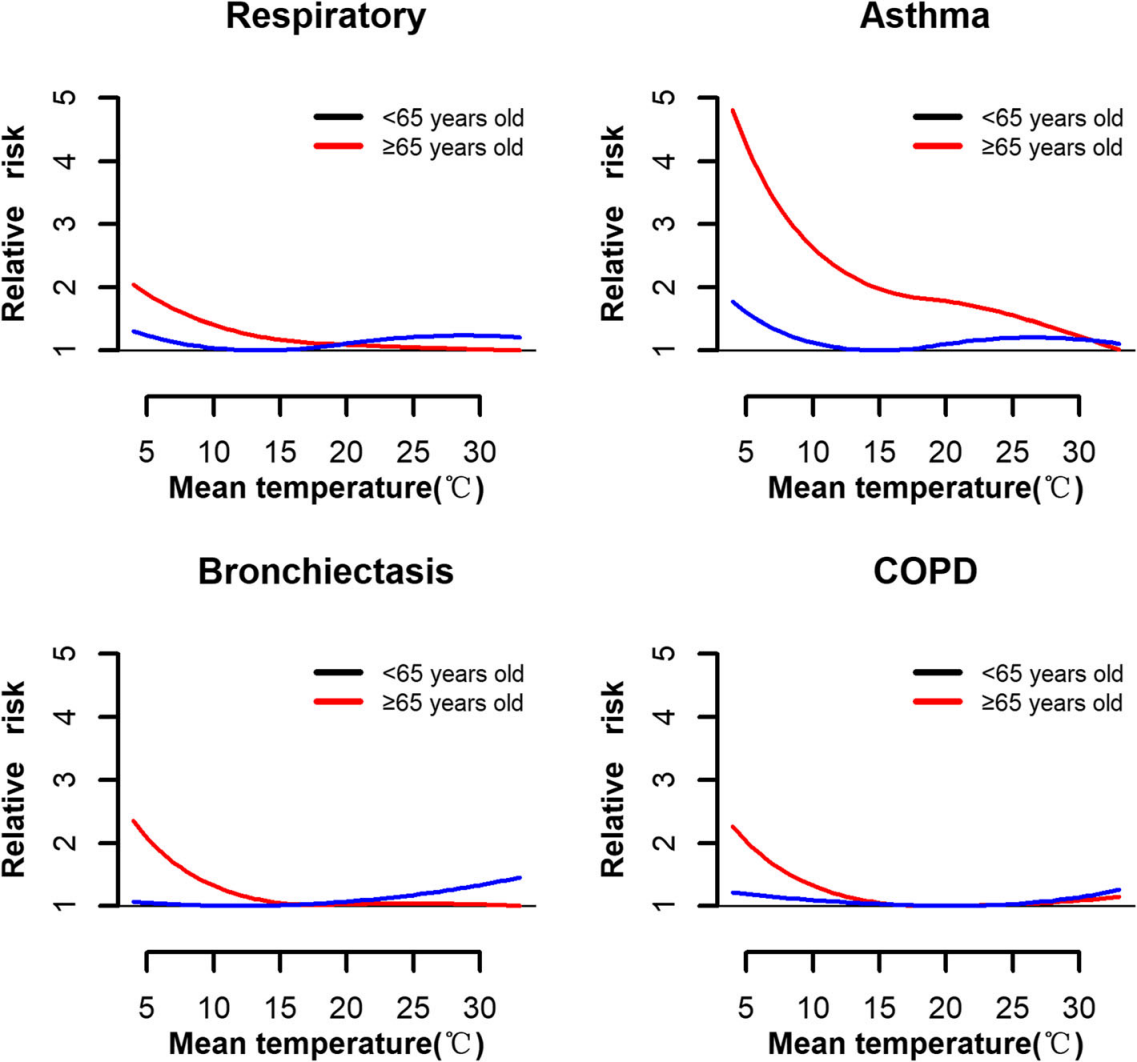
Associations between outpatient visits for total and specific respiratory diseases and daily mean temperatures relative to age group

The risks of extreme temperature on morbidity for total and specific respiratory diseases are displayed in Table 2. Compared with optimum temperature, the overall RRs associated with extreme heat were larger than those associated with extreme cold for total respiratory morbidity, asthma, and bronchiectasis. The effects of extreme cold or heat on outpatient visits for COPD were similar. Figure 3 further presents the temperature-respiratory morbidity relationships by age group. Among younger patients (0–64 years old), the risk of heat on total respiratory morbidity was higher than that of cold. However, a reverse J-shaped relationship was observed for the elderly; namely, the risk of cold on total respiratory morbidity was significantly higher than that of heat. Exposure-response curves for bronchiectasis and COPD followed a similar pattern as total respiratory morbidity. However, for asthma, the cold-related risk was higher than the hot-related risk.

**Table 2:** Relative risk (with 95% CI) of extreme cold and hot temperatures on respiratory outpatient visits at lag 0–7 days in Dongguan city, China

Diseases	Relative risk	
	Extreme cold	Extreme heat
Total respiratory	1.02 (0.99, 1.05)	1.13 (1.01, 1.26)
COPD	1.12 (1.01, 1.24)	1.11 (0.91, 1.37)
Bronchiectasis	1.02 (0.95, 1.09)	1.24 (0.96, 1.60)
Asthma	1.04 (1.00, 1.08)	1.09 (0.92, 1.29)

The extreme cold is the fifth percentile of temperature (12.6 °C), and the extreme heat is the 95th percentile of temperature (30.3 °C), compared with the optimal temperature at a lag of 0–7 days

### Attributable fraction of temperature on respiratory morbidity

Table 3 shows the attributable fraction (%) of outpatient visits for respiratory diseases due to different temperature components. Overall, 8.4% (95% eCI: 2.8–13.3%) of respiratory morbidity was attributed to non-optimal temperatures, and most of these were from moderate heat (7.5, 95% eCI: 2.4–12.2%), and only 0.6% (0.0 to 1.1%) were related to extreme heat. In addition, the burden of respiratory morbidity attributable to extreme cold and mild cold were very small: 0.3% (0.0–0.6%) for extreme cold and 0.0% (0.0–0.1%) for moderate cold. Further results on the attributable number of total and specific respiratory diseases morbidity due to different temperature components are shown in Additional file 3: Table S1.

**Table 3:** Attributable fraction (%) of hospital outpatient visits attributable to ambient temperature exposure for respiratory diseases and subcategories by age-group, 2013 to 2017

Subgroup	Total visits	Overall	Extreme cold	Moderate cold	Moderate heat	Extreme heat
		AF (%)	AF (%)	AF (%)	AF (%)	AF (%)
Total respiratory	75,015	8.4 (2.8, 13.3)	0.3 (0.0, 0.6)	0.0 (0.0, 0.1)	7.5 (2.4, 12.2)	0.6 (0.0, 1.1)
< 65 years	57,567	13.6 (7.3, 19.2)	0.2 (−0.1, 0.4)	0.0 (0.0, 0.0)	12.6 (6.8, 17.8)	1.0 (0.4, 1.6)
≥65 years	17,448	7.5 (−12.7, 23.7)	1.8 (0.1, 3.0)	5.8 (−13.8, 21.1)	0.0 (0.0, 0.0)	0.0 (−0.1, 0.1)
COPD	11,068	5.3 (−2.5, 11.9)	1.1 (0.3, 1.8)	0.7 (−0.7, 1.9)	2.9 (−4.4, 9.0)	0.6 (−0.7, 1.5)
< 65 years	3918	5.5 (−5.4, 13.7)	0.4 (−0.9, 1.4)	0.5 (− 2.2, 2.9)	3.6 (−6.2, 11.8)	0.7 (−1.2, 2.0)
≥65 years	7150	5.4 (−5.3, 14.2)	1.5 (0.6, 2.3)	0.8 (−0.4, 1.9)	2.5 (−7.8, 11.1)	0.3 (−1.3, 1.5)
Bronchiectasis	7991	10.2 (−3.3, 21.0)	0.3 (−0.4, 0.9)	0.0 (−0.2, 0.3)	8.8 (−3.3, 18.8)	1.2 (− 0.3, 2.4)
< 65 years	6113	14.0 (−3.9, 27.9)	0.0 (− 0.3, 0.3)	0.0 (0.0, 0.0)	12.5 (−4.2, 25.5)	1.7 (− 0.3, 3.1)
≥65 years	1878	4.5 (−9.7, 16.7)	0.6 (−5.3, 3.7)	2.0 (−4.0, 3.9)	0.0 (0.0, 0.0)	0.0 (−1.2, 1.0)
Asthma	28,059	8.8 (0.2, 16.1)	0.6 (0.2, 1.0)	0.1 (0.0, 0.3)	7.8 (0.2, 14.5)	0.3 (−0.6, 1.2)
< 65 years	24,808	12.3 (3.4, 19.8)	0.5 (0.0, 0.9)	0.1 (−0.1, 0.2)	11.1 (3.3, 18.1)	0.7 (−0.3, 1.6)
≥65 years	3251	36.7 (5.6, 56.7)	4.3 (1.4, 5.9)	33.7 (2.3, 53.3)	0.0 (0.0, 0.0)	0.7 (−0.1, 1.4)

Moderate cold and heat are temperature ranges between MMT and the 5th or 95th percentile of temperatures, respectively; Extreme cold and heat are temperature ranges at 5th percentile of temperature and below, or at the 95th percentile of temperature and above, respectively

The attributable fractions (%) varied among age-groups (Table 3). For the population aged 0–64 years old, 12.6% of total respiratory, 12.5% of bronchiectasis, and 11.1% of asthma morbidity resulted from exposure to moderate heat. Nevertheless, for the elderly (≥65 years old), the temperature-related morbidity burdens for total and specific respiratory diseases were mainly caused by cold.

## Discussion

In this study, we examined the association between ambient temperature and respiratory morbidity, and further quantified morbidity burden attributed to different temperature ranges in Dongguan city. Our results revealed that a significant percentage (8.4%) of respiratory outpatient visits were attributable to ambient temperatures within the study period. Most of the morbidity burden of total respiratory diseases was caused by moderate heat exposure (7.5%). In general, this finding was consistent with several previous studies [7, 26, 27]. Our results might be explained by the fact that most of days (about 82.2%) within our study time series featured moderate heat. However, our results were different from previous results that were estimated by mortality [28–30]. The difference may be explained mainly by that minimum morbidity temperature is much lower in our study than that minimum mortality temperature identified in previous studies.

We also observed that both cold and heat could increase the risk of respiratory outpatient visits, in agreement with previous studies [7, 20, 31]. However, the mechanisms behind this relationship are not entirely clear. Previous laboratory and clinical studies found that temperatures directly influenced respiratory morbidity by inciting vascular changes, releasing inflammatory mediators, and decreasing the effectiveness of immune responses [32–36]. Moreover, ambient temperatures can indirectly induce respiratory events, such as viral infections, bacterial activity, or respiratory tract infections [37–39].

Stratified analyses by age group showed that younger people (< 65 years) were at high risk of exposure to heat while the elderly (≥65 years) were at high risk of exposure to cold. Moreover, morbidity burdens of total and specific respiratory diseases differed by age group. For younger people, the morbidity burden of total and specific respiratory diseases was mainly attributable to moderate heat. Similar results were also reported before [40]. There are two possible reasons for the observed findings. First, attributable fractions are not only determined by relative risk, but also by temperature distributions. In subtropical cities like Dongguan, most throughout the year are hot days. Younger people tend to stay outdoors longer for work, and are more likely to be exposed to hot ambient temperatures, compared to the elderly. Second, for the younger population, the minimum morbidity temperatures are relatively low for total and specific respiratory diseases. This causes some low temperatures to be classified as hot temperature range, which leads to hot days accounting for a high proportion of our study period data. Therefore, morbidity burdens attributed to heat in the younger population may be very high.

On the contrary, we found that, for the elderly, the attributable fractions of total respiratory diseases, bronchiectasis, and asthma were caused by cold temperatures. The possible reasons are that respiratory diseases are highly prevalent among the elderly, and exposure to cold temperatures may increase the risk of developing pulmonary vascular resistance and thromboses [41, 42]. Also, airway neutrophils, macrophages, and the levels of respiratory inflammation were increased in patients residing in cold environments. Thus, bronchospasm was easily aggravated in patients with asthma, and airway obstruction was easily aggravated in COPD [43].

Our findings have important public health implications because, although extreme heat is riskier than moderate heat, days with moderate heat account for most days in a year in subtropical regions like Dongguan city. Thus, more attention should be paid to moderate heat for the young population when planning adaptation strategies and measures to reduce health risks related to ambient temperature exposures in subtropical regions. Meanwhile, we should put emphasis on the elderly in winter to prevent them from cold exposure.

There were some limitations to the current study. First, this was ecological research which lacked individual-level data pertaining to exposure and the influence of personal activity patterns, etc. Second, we did not obtain epidemiological data pertaining to influenza, which could be a confounding factor or effect moderator. Additionally, hospital visit data were gathered from one single hospital, which may limit the generalizability of our research findings.

## Conclusions

Both high and low temperatures increased the morbidity risk of respiratory diseases. Moderate heat was mainly responsible for most of the morbidity burden caused by ambient temperature exposure. Younger individuals were at increased risk following exposure to hot temperatures, whereas the elderly was more susceptible to cold temperatures. These findings carry implications for planning public health interventions that protect vulnerable populations within non-optimal temperature environments.

## Supplementary information

**Additional file 1: Figure S1.** Geographical distribution of the Dongguan in Guangdong Province, China (the left-upper panel shows the location of Guangdong Province in China). The red spots represent the location of five air monitoring stations, the blue “H” shape Icon represent the location of the Dongguan Fifth People’s Hospital.

**Additional file 2: Figure S2.** The definition of temperature range.

**Additional file 3: Table S1.** Attributable number of hospital outpatient visits attributable to ambient temperature exposure for respiratory diseases and subcategories by age-groups, 2013 to 2017.

## Data Availability

Please contact the author for data requests.

## Abbreviations

AF: Attributable fraction
AN: Attributable number
AR: Attributable risk
CI: Confidence Interval
CO: Carbon monoxide
COPD: Chronic obstructive pulmonary disease
DLNM: Distributed lag non-linear model
eCIs: Empirical confidential intervals
MMT: Temperature of the minimum morbidity
NO_2_: Nitrogen dioxide
PM_10_: Particulate matter with an aerodynamic diameter ≤ 10 μm
QAIC: Akaike Information Criteria for Quasi Poisson
RR: Relative risk
SO_2_: Sulfur dioxide
