# Supplementary figures and images for: Morbidity burden of respiratory diseases attributable to ambient temperature: a case study in a subtropical city in China
Source: Environ Health. 2019 Oct 24;18:89. doi: 10.1186/s12940-019-0529-8 (PMC6814053; doi:10.1186/s12940-019-0529-8)

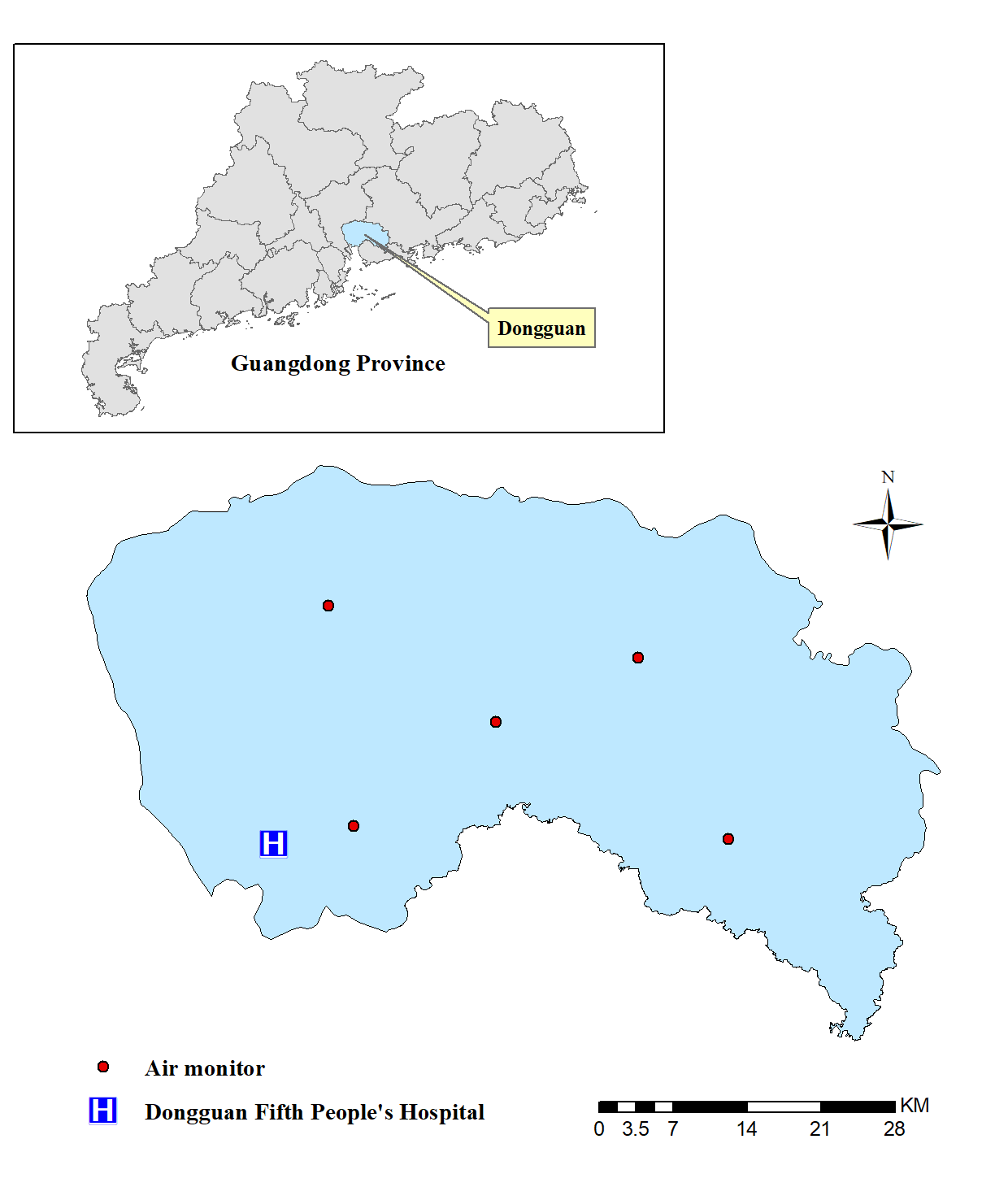

Supplement: Supplementary file 1 — Additional file 1: Figure S1. Geographical distribution of the Dongguan in Guangdong Province, China (the left-upper panel shows the location of Guangdong Province in China). The red spots represent the location of five air monitoring stations, the blue “H” shape Icon represent the location of the Dongguan Fifth People’s Hospital. [file 12940_2019_529_MOESM1_ESM.tif]

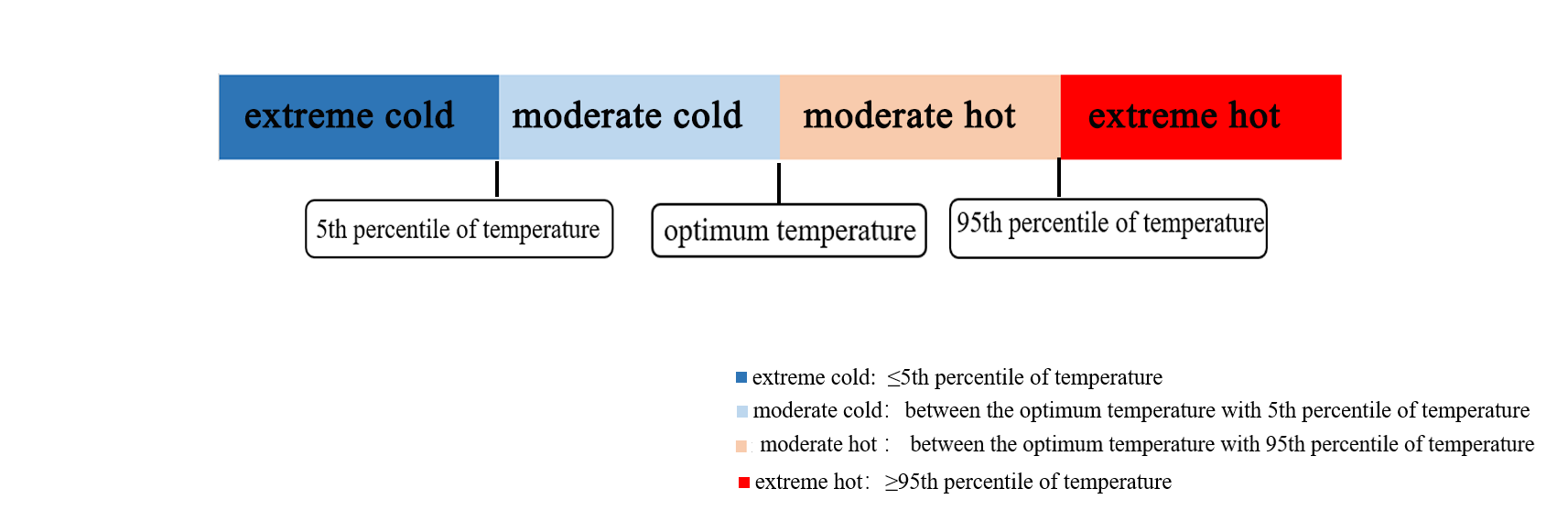

Supplement: Supplementary file 2 — Additional file 2: Figure S2. The definition of temperature range. [file 12940_2019_529_MOESM2_ESM.tif]
