# Supplementary material for: Morbidity burden of respiratory diseases attributable to ambient temperature: a case study in a subtropical city in China
Source: Environ Health. 2019 Oct 24;18:89. doi: 10.1186/s12940-019-0529-8 (PMC6814053; doi:10.1186/s12940-019-0529-8)
Supplement: Supplementary file 3 — Additional file 3: Table S1. Attributable number of hospital outpatient visits attributable to ambient temperature exposure for respiratory diseases and subcategories by age-groups, 2013 to 2017. [file 12940_2019_529_MOESM3_ESM.docx]

Table S1. Attributable number of hospital outpatient visits attributable to ambient temperature exposure for respiratory diseases and subcategories by age-groups, 2013 to 2017.

| Subgroup | Total visits | Overall | Extreme cold | Moderate cold | Moderate hot | Extreme hot |
| --- | --- | --- | --- | --- | --- | --- |
|  |  | AN | AN | AN | AN | AN |
| **Total respiratory** | 75015 | 6265(2163,10059) | 249(26,449) | 32(-28,91) | 5599(1896,9239) | 446(-11,868) |
| <65 years | 57567 | 7852(4376,11184) | 94(-55,231) | 2(-7,11) | 7265(3942,10399) | 593(192,942) |
| ≥65 years | 17448 | 1306(-2234,4093) | 307(21,537) | 1017(-2330,3686) | 0(0,0) | 8(-65,74) |
| **COPD** | 11068 | 588(-268,1290) | 126(38,197) | 77(-73,214) | 317(-487,1008) | 63(-71,172) |
| <65 years | 3918 | 214(-206,533) | 17(-36,56) | 19(-84,112) | 140(-264,467) | 26(-49,80) |
| ≥65 years | 7150 | 385(-391,1019) | 108(40,160) | 57(-28,135) | 181(-538,772) | 21(-95,109) |
| **Bronchiectasis** | 7991 | 813(-233,1678) | 23(-34,71) | 4(-15,23) | 702(-244,1463) | 97(-28,195) |
| <65 years | 6113 | 854(-211,1693) | 1(-21,17) | 0(0,0) | 764(-292,1571) | 102(-17,189) |
| ≥65 years | 1878 | 85(-175,310) | 12(-1,32) | 38(-248,352) | 0(0,0) | 0(-21,18) |
| **Asthma** | 28059 | 2462(123,4504) | 173(45,285) | 30(-12,72) | 2180(26,4088) | 96(-176,318) |
| <65 years | 24808 | 3041(966,4932) | 126(12,226) | 16(-12,44) | 2752(800,4495) | 179(-67,381) |
| ≥65 years | 3251 | 1193(221,1837) | 138(40,192) | 1094(127,1732) | 0(0,0) | 24(-3,47) |

The moderate cold and hot are temperature ranges between MMT and 5th percentile or 95th percentile of temperatures;
The extreme cold and heat are temperature ranges at 5th percentile of temperature and below, or at 95th percentile of temperature and above, respectively
